# Plant geographic phenotypic variation drives diversification in its associated community of a phytophagous insect and its parasitoids

**DOI:** 10.1186/s12862-018-1239-5

**Published:** 2018-09-04

**Authors:** Hui Yu, Dan Liang, Enwei Tian, Linna Zheng, Finn Kjellberg

**Affiliations:** 1 Guangdong Provincial Key Laboratory of Applied Botany, and Key Laboratory of Plant Resources Conservation and Sustainable Utilization, South China Botanical Garden, the Chinese Academy of Sciences, Guangzhou, 510650 China; 2 CEFE, UMR 5175, CNRS, Univ Montpellier, Univ Paul-Valéry Montpellier, EPHE, IRD, 1919 route de Mende, F-34293 Montpellier Cédex 5, France

**Keywords:** Community, Diversification, Co-evolution, *Ficus hirta*, Fig wasp, Ovipositor length

## Abstract

**Background:**

While the communities constituted by phytophageous insects and their parasites may represent half of all terrestrial animal species, understanding their diversification remains a major challenge. A neglected idea is that geographic phenotypic variation in a host plant may lead to heterogeneous evolutionary responses of the different members of the associated communities. This could result in diversification on a host plant by ecological speciation in some species, leading to geographic variation in community composition. In this study we investigated geographic variation of inflorescence receptacle size in a plant, *Ficus hirta*, and how the hymenopteran community feeding in the inflorescences has responded. Our predictions were:

1. Inflorescence size variation affects wasp species differently depending on how they access oviposition sites.
2. In some affected lineages of wasps, we may observe vicariant, parapatric species adapted to different inflorescence sizes.

**Results:**

We show that fig (the enclosed inflorescence of *Ficus*) wall thickness varies geographically. The fig-entering pollinating wasp was not affected, while the parasites ovipositing through the fig wall were. Two parapatric species of *Philotrypesis*, exhibiting strikingly different ovipositor lengths, were recorded. One species of *Sycoscapter* was also present, and it was restricted, like the shorter-ovipositor *Philotrypesis*, to the geographic zone where fig walls were thinner.

**Conclusions:**

Previous work on fig wasps suggested that parapatric geographic ranges among congenerics were due to adaptation to variation in abiotic factors, complemented by interspecific competition. Our results show that parapatric ranges may also result from adaptation to variation in biotic factors. Within an insect community, differences among species in their response to geographic phenotypic variation of their host plant may result in geographically heterogeneous community structure. Such heterogeneity leads to heterogeneous interaction networks among sites. Our results support the hypothesis that plant geographic phenotypic variation can be a driver of diversification in associated insect communities, and can complement other diversification processes.

**Electronic supplementary material:**

The online version of this article (10.1186/s12862-018-1239-5) contains supplementary material, which is available to authorized users.

## Background

Since the early 1980’s the quest for understanding diversification in the communities of phytophages and their parasites associated with plants, has largely focused on the consequences of the colonisation of a new host by an insect phytophage or a parasitoid and the resulting specialisation process [1]. Such host shifts often lead to the formation of host-races and may trigger a cascade of diversification as parasites and hyper-parasites may follow their phytophagous host. Alternatively, parallel diversification of plant host and their associated parasites may occur. In this process, host plant separation into distinct lineages correlates with associated parasite separation into distinct lineages [2, 3]. Hence there has been an emphasis on exploring the respective roles of parallel diversification versus host shifts in patterns of community diversification [4–7].

An important facet of diversity is represented by geographic variation in its associated communities across the range of a host plant. Widely-distributed plants are likely to be growing in areas with a wide range of environmental conditions, which will vary in suitability for their associated insects. Therefore, large host plant ranges allow the establishment of parapatric vicariant associated insect species, adapted to different environmental conditions [8–10]. In this process, diversification is a response to geographic variation in abiotic factors such as minimum and maximum temperatures [11].

Geographic variation in biotic factors may also strongly affect the interactions between members of the associated communities. If we focus on variation in biological traits of the plants and their associated community member species, then two major processes may be involved. Traits of the interacting species may evolve in direct response to each other, in a co-evolutionary process. However, there is limited support for an important role of coevolutionary diversification in the diversification of life [2, 3]. Alternatively, phenotypic variation in one species may be driven by factors extrinsic to the interaction, but nonetheless result in divergent selection on associated species. Classical examples of structured phenotypic variation in plants are geographic variation in plant phenology [12] and geographic variation in the size of particular plant organs [13]. Such variation in plant traits is either a direct plastic phenotypic response to local conditions or the product of local adaptation. Nevertheless, it may have a strong impact on phytophages and their parasites.

**Fig. 1:**
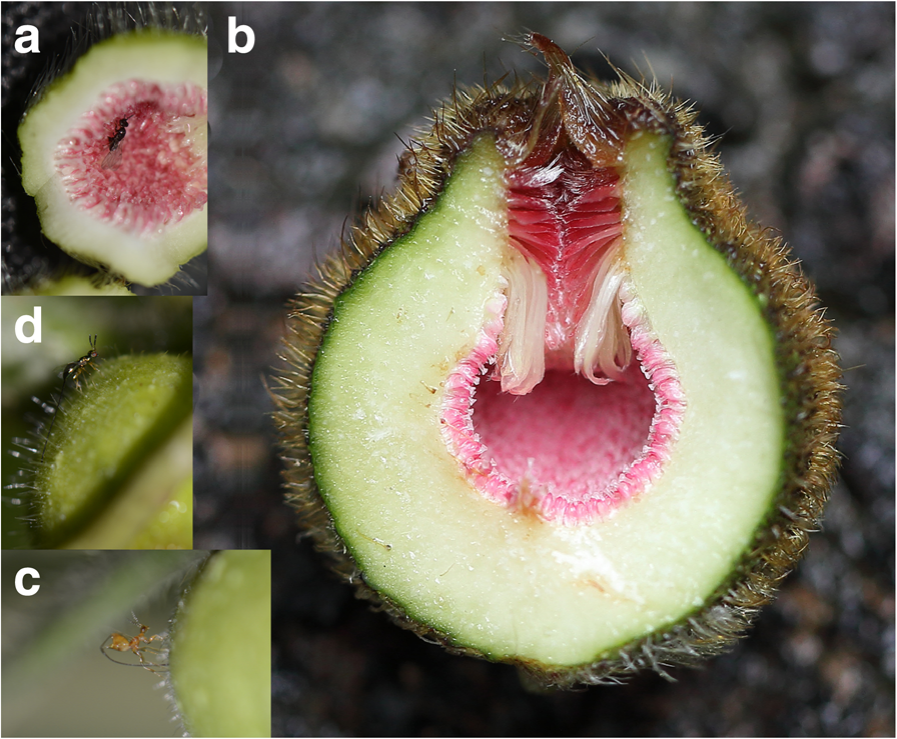
Geographic variation in fig wall thickness and wasp oviposition. **a** A *Ficus hirta* fig from Guangzhou in the south-western part of the study region. A pollinating wasp has entered the fig cavity and is ovipositing into the flowers that line the fig cavity. **b** A *Ficus hirta* fig from Fujian province, in the north-eastern part of the range of the species. Note the larger size of the fig and the thicker fig wall. **c** An ovipositing *Philotrypesis*. The wasp inserts its ovipositor through the fig wall and will only lay an egg if it reaches an ovule colonised by a pollinator larva. **d** An ovipositing *Sycoscapter hirticola.* The wasp inserts its ovipositor through the fig wall and will only lay an egg if it reaches an ovule colonised by a pollinator larva. The thick fig walls in the northern eastern part of the range of *Ficus hirta* represent an evolutionary challenge for the parasitic wasp species which oviposit through the fig wall but not for the pollinator which enters the fig

*Ficus* and fig wasps provide a model system to investigate geographic variation in associated community composition. Indeed, *Ficus* species in general have exceptionally large geographical distributions for tropical trees [14], a feature that facilitates the investigation of geographic variation. Furthermore, sampling whole communities of fig-wasps and assessing specificity is straight-forward. *Ficus* are characterised by their urn-shaped inflorescence called a fig, the inside of which is lined by female flowers (Fig. 1). A female flower may become a seed, or be transformed into a gall by the mutualistic pollinators or by galling wasps. The galls may in turn be attacked by cleptoparasites or parasitoids. Each galled flower finally produces one adult wasp offspring [15]. The communities of non-pollinating fig wasps on different continents or on different subgenera of *Ficus* exhibit strikingly different phylogenetic relationships, yet they have similar functional organization, demonstrating convergent evolution of community structure [15].

Pollinating wasps enter the fig cavity through an ostiole and oviposit into ovules by inserting their ovipositor through flower styles, whereas most parasites oviposit through the fig wall (Fig. 1). Hence ostiole and style modifications affect pollinators while fig wall modifications affect parasites [16]. This provides for a decoupling of constraints on pollinators and parasites and, hence, a system in which decoupling of diversification processes can be investigated. Furthermore, *Ficus* display great intraspecific variation in fig size within species [17]. In some *Ficus* species, this variation is spatially structured [18].

Here we document, in *Ficus hirta*, geographic variation in fig morphology in South-East China and a correlated difference in the composition of its associated parasitic fig-wasp community. This variation occurs within a context of strong gene flow and a lack of genetic isolation by distance, in both *Ficus hirta* and its pollinating wasp *Valisia hilli*, throughout the region [9].

## Methods

### Natural history of system

*Ficus hirta* Vahl is a tropical to subtropical shrub of secondary vegetation. In South-East China it is pollinated by *Valisia hilli* Wiebes (Agaonidae, Chalcidoidea). *Ficus hirta* is a functionally dioecious species. In female plants, flower ovules produce seeds and no wasps, while in functionally male plants, flower ovules host wasp larvae and do not produce seeds. During sampling we observed two parasitic fig wasps, *Philotrypesis josephi* and *Sycoscapter hirticola* (Sycoryctinae, Pteromalidae, Chalcidoidea) [19]. Both species oviposit from outside the fig into ovaries by inserting their long ovipositor through the fig wall (Fig. 1). Wasps of the genus *Philotrypesis* are assumed to be cleptoparasites, each larva replacing a pollinator larva and feeding on gall tissue. Wasps of the genus *Sycoscapter* are assumed to be parasitoids, each larva feeding on one pollinator larva [20]. *Philotrypesis josephi* and *Sycoscapter hirticola* oviposit in figs that have been recently visited by pollinators (Yu, unpublished observations).

### Sites and sample collection

**Fig. 2:**
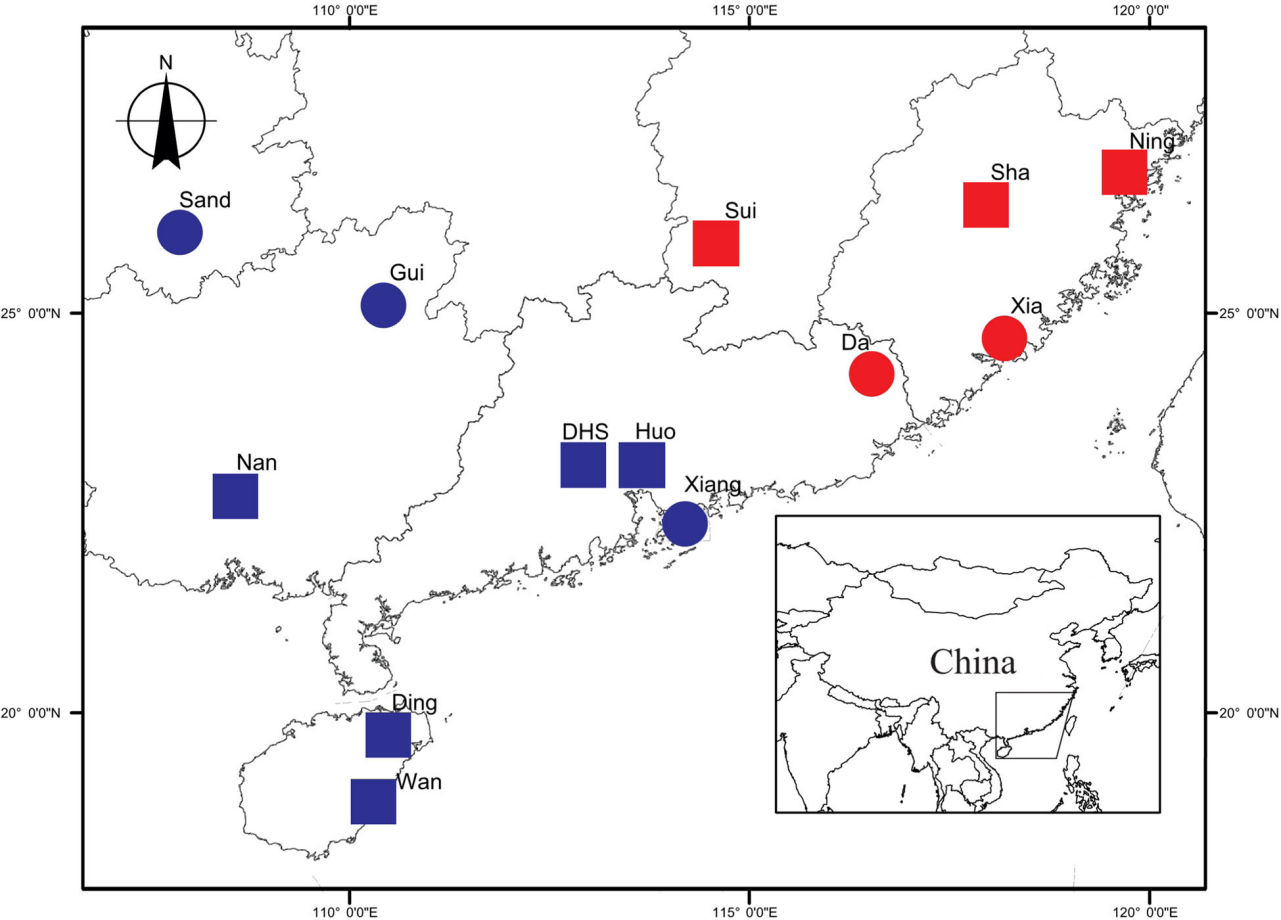
Sampling sites. North-East: red; South-West: blue. All the sites were included in the microsatellite genetic analyses, 8 for fig morphology, for wasp ovipositor length and for wasp COI sequencing. Map template provided by the Chinese National Mapping and Geographic Information Bureau, http://bzdt.nasg.gov.cn

Between June 2006 and July 2013, we sampled wasps for genetic analysis from 11 sites on the mainland of South East China and 2 sites in Hainan island (Fig. 2; Table 1) spanning a distance of over 1000 km, and reaching the north-eastern limit of the range of *F. hirta*. Figs and parasitic wasps were collected for morphological assessment from 8 sites. *Philotrypesis* wasps were present in all sites while *Sycoscapter* wasps were absent from the north-eastern sites. Hence, for *Sycoscapter*, we obtained samples for genetic analysis from 7 sites on the mainland of South East China and 2 sites in Hainan Island, and morphological analysis was carried out for 5 sites.

**Table 1:** Sampling sites and sample sizes for microsatellite analysis

Site	Latitude, Longitude	*Philotrypesis*	*Sycoscapter*
Ning	26°39′51″N, 119° 32′ 56″E	25	*–*
Sha	26°23′50″N, 117° 47′ 32″E	20	*–*
Xia	24°44′33″N, 118°4′20″E	11	*–*
Sui	25°45′09″N, 114° 16′ 58″E	24	*–*
Da	24°15′30″N, 116° 48′22″E	15	–
Gui	25°04′ 36” N, 110°18′21″E	12	8
Huo	23°11′11″N, 113° 21′ 56″E	15	24
DHS	23°02′49″N, 112° 27′ 54″E	16	19
Xiang	22°23′47″N, 114° 06′ 34″E	21	21
Sand	25°59′08″N, 107° 52′ 28″E	12	21
Nan	22°49′03″N, 108° 21′ 56″E	20	9
Ding	19°41′50″N, 110° 19′ 42″E	24	24
Wan	18°47′42″N, 110° 23′ 27″E	17	17
Total		207	148

### Fig size variation and ovipositor length of *Philotrypesis* and *Sycoscapter*

Figs on male trees were collected in January 2015, at the stage at which the parasitic wasps oviposit (the early phase after pollinator oviposition, when the fig cavity is filled up by flowers transformed into galls, each containing a wasp larva). The length and the diameter of figs and the thickness of the fig wall were measured using readings of a Vernier caliper to the nearest hundredth of a millimeter. Wasps were collected following Tian et al. [9]. Wasps preserved in 70% ethanol were dissected to reveal the entire length of the ovipositor and measured under a dissection microscope to the nearest 0.01 mm using a measuring eyepiece. Body length was measured excluding the modified terminal tergites which cover the sheath of the ovipositor.

Size measurements were analyzed using linear models as implemented in R3.3.2 with the package lmtest [21]. The correlations between fig diameter and thickness of the fig wall on one hand, and wasp size, and ovipositor length on the other hand were analyzed using multiple linear regressions comparing north-eastern versus south-western regional effects and individual site effects.

### Species discrimination using sequence data and population genetics

In each site, 10 to 30 mature figs were collected just before wasp emergence. The figs were placed in fine-mesh bags to allow the wasps to emerge naturally. The wasps that emerged were preserved in 95% ethanol and stored at − 20 °C until deoxyribonucleic acid (DNA) extraction. For each species, a single female wasp per fig was used for genetic analyses. Sample sizes are given in Table 1.

To delimit species, we sequenced the cytochrome c oxidase subunit I (COI) gene on a number of individuals of *Philotrypesis* and *Sycoscapter* from every site for which wasp measurements were made, representing a total of 70 individuals from 8 sites for *Philotrypesis* and 73 individuals from 5 sites for *Sycoscapter*. Seven published *Philotrypesis* COI sequences, 3 from Hainan Island and 4 from Fujian were also included in the analysis [22, 23]. We built COI neighbor joining trees for the two genera. Putative species were delimited as clearly defined monophyletic clades in neighbour joining trees. Genetic separation between putative species was visualized by plotting Kimura 2-parameter pairwise genetic distances (K2P) [24] within species and between species. For the purpose of comparison, we plotted the distribution of K2P pairwise distances for the pollinating wasps using previously published data for the same region [9]. To provide a background framework, we analyzed the fine grained spatial genetic structure within *F. hirta*, by plotting genetic differentiation between populations (*F*_*ST*_/1-*F*_*ST*_) (*F*-statistics describe the statistically expected level of heterozygosity in a population, and *F*_*ST*_ is the effect of subpopulations compared to the total population) according to distance using previously published microsatellite genotype data [25].

Genomic DNA was extracted from the whole body of each female fig wasp using the EasyPure Genomic DNA Extraction Kit (TransGen, Beijing, China). A 545 bp fragment of the COI gene of *Philotrypesis* and 560 bp fragment of the COI gene of *Sycoscapter* was amplified using the universal primer pair (LCO1490: 5’-GGTCAACAAATCATAAAGATATTGG-3′, HCO2198: 5′- TAAACTTCAGGGTGACCAAAAAATCA -3′) [26]. PCR amplification of COI was carried out in a 20 μl volume using 10× buffer, 2 mM Mg^2+^, 0.25 mM each dNTP, primer 0.6 uM, Taq polymerase (Takara) 2 U and DNA 150 ng. The reaction was optimized and programmed on a MJ Thermal Cycler (PTC 200) as one cycle of denaturation at 94 °C for 3 min, 35 cycles of 30 s denaturation at 92 °C, 30 s at a 48 °C annealing temperature, and 30 s extension at 72 °C, followed by 10 min extension at 72 °C. All amplified PCR products were purified using QIAquick spin columns (Qiagen) and were sequenced in an ABI 3730xl capillary sequencer using BigDye Terminator V 3.1 chemistry (Applied Biosystems). Consensus sequences were aligned using MUSCLE implemented in MEGA 6.0 [27] with manual corrections. Haplotype sequences have been deposited in GenBank under Accession No: MG548662–700 and MG548701–24.

We used a neighbour-joining tree (NJ tree) to reconstruct the phylogenetic relationships based on all COI sequences of *Philotrypesis* and *Sycoscapter*. NJ was reconstructed using MEGA 6.0 [27] and node supports were assessed based on 1000 bootstrap replicates. For *Philotrypesis*, we also include *Philotrypesis anguliceps* hosted by *Ficus religiosa* (JQ408678), *Philotrypesis* sp1 hosted by *Ficus oligodon* (JN545271), and two haplotypes of *Philotrypesis spinipes* hosted by *Ficus fistulosa* (JQ408674, JQ408677) [22, 23] as the outgroup. For *Sycoscapter*, we use three *Philotrypesis* sequences (one from the south-western species and two from the north-eastern species) generated in the current study as outgroups because of a lack of COI sequences of the family in Genbank. K2P pairwise distances were calculated within species and between putative congeneric species.

In a second step, individuals from all sampling sites were genotyped at microsatellite loci and Bayesian clustering was used to determine the optimal number of groups using STRUCTURE 2.3.4 [28] and STRUCTURE HARVESTER [29]. Assignation of individuals within sites to clusters was compared with the geographic distribution of putative species according to COI genotyping, to detect potential local co-occurrence of species.

Pairwise *F*_*ST*_ values of genetic differentiation among populations were calculated and the values compared using SPAGeDI1.5 [30]. Significance of spatial genetic structure was evaluated with 20,000 permutations.

For *Philotrypesis,* two hundred and seven individuals were genotyped at 6 unlinked microsatellite loci (P50, P86, P91, P123, P129 and P202) that had been previously developed for the species [31], and for *Sycoscapter hirticola*, one hundred and fourty-eight individuals were genotyped at 7 unlinked microsatellite loci (S7, S27, S52, S122, S132, S137 and S162) that had been previously developed for the species [32]. Molecular analysis techniques followed Abellò et al. [31] for *P. josephi* and Ahanchédé et al. [32] for *S. hirticola*.

The methods for PCR amplification and analysis of microsatellites were identical between *Sycoscapter* and *Philotrypesis* excep

t for the annealing temperature. The amplification reactions were conducted using a PTC-200 thermal cycler (Bio-Rad, Hercules, CA) in 20 μl volume containing 20 ng of genomic DNA, 0.2 mM of each dNTP, 0.4 μM of fluorescent primer, 1 ul of 10 × PCR buffer (Mg^2+^ free), 2.5 mM Mg^2+^ and 1 unit of Taq DNA polymerase (Takara, Dalian, China), using the following conditions: initial denaturation at 95 °C for 5 min, 35 cycles of (94 °C, 30 s; 46 to 50 °C, 60 s for *Sycoscapter* and 72 °C, 45 s; 47 to 52 °C, 60 s for *Philotrypesis*) and a final extension of 72 °C for 8 min (see Table 1 for annealing temperatures of each primer pair). The fragment sizes of the PCR products were determined on the ABI PRISM 3100 Genetic Analyser (Applied Biosystems, Foster City, CA) using genotyper 4.0 and LIZ 500 (Applied Biosystems, Foster City, CA) as an internal size standard.

## Results

**Fig. 3:**
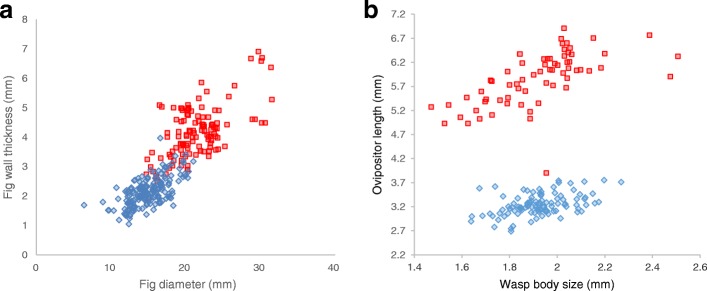
Fig size, fig wall thickness and *Philotrypesis* ovipositor length. **a** Figs from north-eastern sites (red squares), are large and as a consequence have thicker fig walls than figs from south-western sites (blue diamonds). **b** While wasps from south-western sites (blue) and the wasps from north-eastern sites (red) exhibit identical body lengths, they differ strongly in the relationship between body size and ovipositor length

Fig wall thickness varied geographically and was correlated with the presence and morphology of the parasitic wasps. *Ficus hirta* individuals from north-eastern sites produced figs with thicker walls than individuals from south-western sites (F_1,288_ = 970, *p* < 10^− 93^). The difference was mainly due to the production of larger diameter figs, which exhibited thicker fig walls (Fig. 1; Fig. 3; Table 2). Indeed, the correlation of fig wall thickness with fig size explained 70% of the variance in fig-wall thickness (F_1,288_ = 1031, *p* < 10^− 96^). A north east-south west difference in the correlation was also detected, but explained only 8.8% of the variance (F_1,288_ = 127, *p* < 10^− 24^).

**Table 2:** Fig and wasp morphology according to sampling site

	Fig			*Philotrypesis*		*Sycoscapter*	
Site	figs /plants	Fig diameter (mm)	Thickness of fig wall (mm)	wasps	Ovipositor length (mm)	wasps/figs	Ovipositor length (mm)
Northern sites							
Ning	36/9	23.23 ± 4.87	4.91 ± 0.89	24/10	5.99 ± 0.42	0/0	
Sha	43/14	21.42 ± 2.62	3.88 ± 0.59	21/6	5.73 ± 0.32	0/0	
Sui	42/12	19.99 ± 2.62	3.74 ± 0.79	23/5	5.31 ± 0.43	0/0	
Southern sites							
Huo	42/21	14.32 **±** 2.75	1.92 ± 0.40	21/3	3.31 ± 0.23	14/5	3.55 ± 0.24
DHS	40/16	13.39 **±** 1.72	2.10 ± 0.34	21/8	3.24 ± 0.24	13/7	3.52 ± 0.23
Nan	41/13	16.76 **±** 2.04	2.38 ± 0.43	21/6	3.43 ± 0.23	11/5	3.23 ± 0.13
Ding	14/9	15.47 **±** 1.64	2.34 ± 0.36	21/4	3.12 ± 0.12	4/2	3.21 ± 0.19
Wan	33/11	15.32 **±** 2.26	2.12 ± 0.70	20/4	3.16 ± 0.15	11/2	3.25 ± 0.13

*Philotrypesis* wasps and *Sycoscapter* wasps exhibited similar ovipositor length at south-western sites, with a mean value per site of 3.12–3.31 mm for *Philotrypesis* and 3.21–3.55 mm for *Sycoscapter* (Fig. 3; Table 2). In the north-east, *Sycoscapter* was totally absent, while *Philotrypesis* wasps exhibited much longer ovipositors, three times as long as body length (Fig. 3; mean value per site: 5.31–5.99 mm, Table 2). At all sites, the average length of parasite ovipositors was larger than the average thickness of fig wall, allowing oviposition to take place (Table 2). Ovipositors of wasps from south-western sites were all shorter than fig-wall thicknesses in north-eastern sites, preventing reproduction of potential south-north migrants (Table 2). While *Philotrypesis* wasps in north-eastern sites exhibited much longer ovipositors than in south-western sites (F_1,167_ = 1824, *p* < 10^− 90^), wasp body size was not larger (Fig. 3, F_1,167_ = 0.0086, *p* = 0.93). The difference resulted from a major difference in mean value of ovipositor length, which explained 90% of the variance, and to a lesser extent from a difference in the slope of the correlation between body length and ovipositor length. Although ovipositor length was correlated with wasp length in both *Philotrypesis* wasps and *Sycoscapter* wasps, for *Philotrypesis* wasps the slope of the correlation was steeper among north-eastern wasps than south-western wasps (Fig. 3, F_1,165_ = 7.97, *p* = 0.005;).

**Fig. 4:**
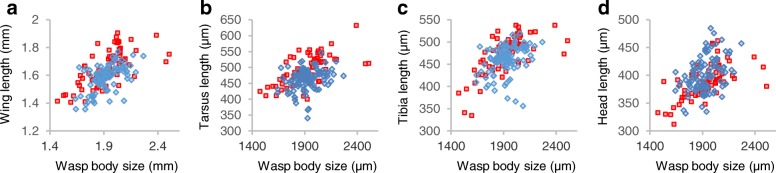
Allometric relationships in *Philotrypesis*. **a** Wing length versus body length. **b** Tarsus length versus body length. **c** Tibia length versus body length. **d** Head length versus body length. Northern wasps: red squares; southern wasps: blue diamonds. Northern wasps exhibited modified allometry with longer wings and tarsi than southern wasps while tibia and head lengths were similar

*Philotrypesis* wasps also exhibited variation in other body traits. Tibia length and femur length were slightly longer in north-eastern than in south-western wasps (the difference representing respectively 2.5 and 3.7% of the variance, *p* < 0.05), while the tarsi of north-eastern wasps were markedly longer (Fig. 4b, representing 14% of the variance, F_1,167_ = 27.7, *p* < 10^− 6^;). North-eastern wasps also exhibited disproportionally longer wings (Fig. 4a, the geographic regions representing 10% of the variance, F_1,165_ = 19.2, *p* < 10^− 4^). On the other hand head size differed only slightly between north-eastern and south-western wasps (Fig. 4d, explaining 3% of the variance, F_1,166_ = 5.05, *p* < 0.05). Hence, the difference in ovipositor allometry correlated with differences in the allometries of a series of traits such as wing length and tarsus length. Site effects within region were generally significant, but explained much less of the variance than the binary geographic effect.

**Fig. 5:**
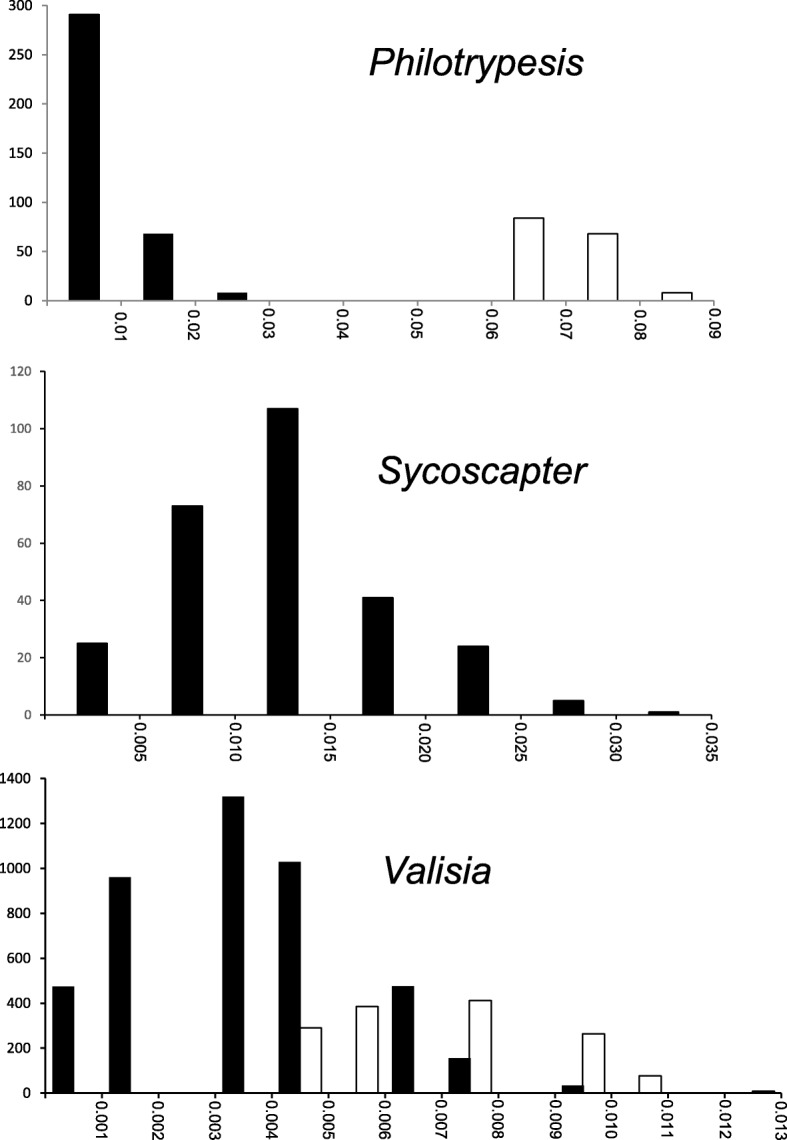
K2P pairwise genetic distances within genus for COI sequences. For *Philotrypesis*, in black comparisons within morphotype, in white comparisons between morphotypes. For *Valisia*, in white comparisons between individuals originating from the continent versus Hainan Island

Putative species were delimited as clearly defined monophyletic clades in neighbour joining trees based on COI data. Haplotype differentiation was limited in *Sycoscapter,* suggesting the presence of a single species (Additional file 1: Figure S1). This result was confirmed by a unimodal distribution of K2P distances between individuals (Fig. 5). Haplotype differentiation was much higher in *Philotrypesis*, clearly separating the two putative species (Additional file 1: Figure S2). All sequenced individuals from short ovipositor populations belonged to one putative species and all sequenced individuals originating from long ovipositor populations belonged to the other putative species (100% branch support). The K2P pairwise distance distribution shows a marked barcoding gap separating within and between putative species pairwise distances (Fig. 5). K2P pairwise distances within the pollinator *Valisia hilli* were much smaller than for *Philotrypesis* and *Sycoscapter* (Fig. 5). The distribution of K2P pairwise distances on the continent was unimodal, with no separation between individuals originating from north-eastern or south-western locations. Finally, in the pollinator, the distribution of K2P pairwise distances between the continent and Hainan Island was displaced towards somewhat higher values, but without a barcoding gap (Fig. 5).

For *Sycoscapter*, the optimal number of genetic groups following the Evanno criterion [33] was one or two. For K = 2, STRUCTURE results separated the closely located sites Huo, Xiang and DHS from the other sites, suggesting a geographic effect (Additional file 1: Figure S3a).

Running STRUCTURE with *Philotrypesis* microsatellite genotypes for K = 2 separated individuals into those originating from north-eastern populations and those originating from south-western populations, supporting the separation of *Philotrypesis* into two allopatric putative species (Additional file 1: Figure S3b). According to the Evanno criterion, the optimal value of K was 4. One cluster was constituted by the north-eastern species, and the south-western species was distributed into three groups that were not separated by the COI sequences (Additional file 1: Figure S3c).

In the short ovipositor *Philotrypesis* putative species, there was no genetic isolation by distance and genetic differentiation among sites was high (Additional file 1: Figure S4; global *F*_*ST*_ value = 0.22). In the long ovipositor *Philotrypesis* putative species, genetic differentiation among sites was much more limited ((Additional file 1: Figure S4; global *F*_*ST*_ value = 0.034) and there was also no genetic isolation by distance. In the *Sycoscapter* species, differentiation among sites was intermediate (Additional file 1: Figure S4; global *F*_*ST*_ value = 0.13) and there was significant isolation by distance (*p* = 0.01). In both *Philotrypesis* and *Sycophaga*, Hainan Island did not show any disproportionately greater genetic differentiation compared to continental populations. In *Valisia*, there was limited genetic differentiation among continental sites, and the variation was not spatially structured (Additional file 1: Figure S4).

Gene diversity values for microsatellite data suggest that the short ovipositor *Philotrypesis* putative species is genetically more diverse than the long ovipositor putative species (Nei gene diversity corrected for sample size [34], short He = 0.84; long He = 0.68; *Sycoscapter* He = 0.64; pollinator He = 0.67, recalculated from previously published data).

For *F. hirta*, genetic differentiation according to distance for continental sites separates a very homogeneous group of sites including all analyzed south-western sites plus site Da. The three other analyzed north-eastern sites are more different, between themselves and comparatively to all other sites, with no effect of geographic distance (Additional file 1: Figure S4).

## Discussion

Our results show that figs of *F. hirta* have thicker walls in north-eastern sites than in south-western ones. In south-western sites we observed one species of *Sycoscapter* and one species of *Philotrypesis*. Both species exhibited relatively short ovipositors that could only allow them to oviposit in south-western figs. In north-eastern sites, *Sycoscapter* was absent and *Philotrypesis* was represented by a distinct lineage exhibiting long ovipositors that allow it to oviposit into north-eastern figs. In parasitic fig wasps, such differences in ovipositor length are generally observed in comparisons between species ovipositing at markedly different times in fig development and hence in host larval development [8, 15, 35]. To our knowledge, it has never been documented between vicariant lineages on the same host.

Longer ovipositors generate a number of morphological constraints on the wasps. During oviposition of *Philotrypesis*, the ovipositor shaft is inserted orthogonally to the fig surface (Fig. 1c) and hence, the use of longer ovipositors requires longer legs, as we observed, mainly due to longer tarsi. Further, an ovipositor twice as long as body size is an impediment to flight and this may explain why north-eastern *Philotrypesis* exhibited longer wings compared to body size.

The ovipositor length of externally-ovipositing non pollinating fig wasps often limits the number of hosts accessible to them. In some cases this is due to variation in fig wall thickness that totally protects some figs against parasites [16]. In other cases those galls containing potential hosts, that are located furthest away from the fig wall are out of the reach of the parasite’s ovipositors [36, 37]. This suggests a trade-off between costs and benefits of longer ovipositors. Our results suggest that one of the costs could relate to flight capacity.

In *Philotrypesis*, the distribution of K2P pairwise distances was bimodal with a marked barcoding gap, suggesting the presence of two distinct species. Results obtained for nuclear microsatellite data using STRUCTURE for K = 2 were consistent with a subdivision into two distinct genetic entities. Non ambiguous genetic separation correlating with large morphological difference between the two morphs shows that the two *Philotrypesis* lineages are distinct species.

Bayesian assignment using STRUCTURE for values higher than 2 allowed separating the short ovipositor *Philotrypesis* genotypes originating from different sites into different clusters. This structuring among south-western sites is confirmed by high *F*_*ST*_ values and represents genetic differentiation within species, among sites. This differentiation did not follow a pattern of genetic isolation by distance. On the other hand, in *Sycoscapter*, the assignation to cluster using STRUCTURE followed a geographic proximity pattern, suggesting genetic isolation by distance. This interpretation is further supported by a significant correlation between geographic distance and genetic differentiation between populations, *F*_*ST*_/(1-*F*_*ST*_). Finally, in *Valisia*, there was limited, unstructured variation on the continent. However, *Valisia* wasps from Hainan Island were clearly differentiated [9] while such a differentiation was not observed in *Philotrypesis* and *Sycophaga*. Hence the patterns of geographic genetic variation were strikingly different among the four wasp species associated with figs of *Ficus hirta.* Heterogeneous spatial genetic structure among the different members of the associated wasp community has also been reported for other fig wasps communities [10]. Such differences among members of a community may lead to heterogeneous rates of speciation.

The presence of two *Philotrypesis* species on *Ficus hirta* could result either from speciation on *F. hirta* or from a host shift, where one of the species originated from another *Ficus* species. In the study zone, and in China in general, the only close relative of *Ficus hirta* is *Ficus triloba* [39]. In our collections of wasps from *Ficus triloba* we have never observed any *Philotrypesis*, but we have collected a species of *Sycoscapter* with a very long ovipositor. This suggests that *Philotrypesis* has speciated on *Ficus hirta*, a proposition that needs to be confirmed by a broader phylogenetic study of the *Philotrypesis* species associated with *Ficus* section *Eriosycea*. On the other hand *Sycoscapter* has failed to colonize north-eastern sites, suggesting limiting adaptive potential.

We may speculate as to which evolutionary process led to speciation in *Philotrypesis*. A classic scenario would be based on an allopatric diversification process made possible by fragmentation of the range of the associates during climatic oscillations. This would have allowed local (co)evolution in isolation, resulting in differentiated morphs in the plant, and speciation in *Philotrypesis*. However, almost all available genetic data on *Ficus*, including *Ficus hirta*, evidence strong gene flow and limited, if any, genetic structuring for neutral genes across continental ranges [25, 40, 41]. This suggests that genetic differentiation in isolation is infrequent within *Ficus* species. Hence, for *Ficus* associated insect communities, evolutionary scenarios that do not involve isolated plant populations may be more realistic. Whatever the initial process leading to insect speciation, data on pollinator and parasite communities, have evidenced geographically structured patterns of insect species replacement, within a context of continuous *Ficus* populations [8, 38, 41]. We favour a scenario in which the fig host was never geographically isolated but developed geographically structured morphological variation involving a thicker wall fruit. This did not pose a challenge to the fig entering pollinator as evidenced by the lack of spatial genetic structure of *Valisia* across continental sites. But only one of the parasitic wasp species followed suite and morphologically differentiated to adapt to the thicker wall of the fig in the north. A process which may have been facilitated by assortative mating, as female wasps born in thick walled figs can only mate with males borne in the same figs, i.e. by offspring of female wasps having long ovipositors.

Previous analysis of *F. hirta* has indicated that the fig lacks genetic population structuring across our study area in continental China (Additional file 1: Figure S4) [9]. Therefore, morphological variation in fig wall thickness could be due to a phenotypic response to local conditions without genetic variation. Alternatively the variation could be adaptive and genetically based. Variation in fig size may have been selected for a series of reasons other than protection against parasitism. For instance, low temperatures increase wasp development time [42]. Large figs automatically become warmer than small ones due to the thicker boundary layer of larger objects, limiting heat dissipation. As a consequence large figs often require strong evaporation to control their temperature [42]. This physical constraint may explain the absence of large fig *Ficus* species in dry tropical environments [43]. In more temperate environments such as in the north-eastern part of *F. hirta*’s distribution, larger figs will warm up more rapidly in the sun, and a thicker fig wall would buffer daily temperature variations, accelerating pollinator development in cool seasons. Hence larger figs and thicker fig walls could have been selected in *Ficus hirta* for their effect on the speed of development of pollinating wasp. In agreement with this hypothesis, in *Ficus hirta*, figs are larger on male plants (producing pollinating wasps and pollen in their figs) than in female plants (producing seeds in their figs) [44]. An alternative could be selection on another phenotypic trait, such as leaf size, indirectly affecting fig size due to ontogenic size correlations between different plant organs [45], although we failed to detect variation in leaf size in our herbarium samples, arguing against the ontogenic hypothesis. Finally, selection of thicker fig walls could also be due to parasitic pressure, in a coevolutionary process. Indeed, a study in the south-western zone has shown that parasites had a strong impact on pollinator wasp mortality, as they killed at least 18% of the pollinating wasp larvae [37]. Further, developing larvae located closest to the figs wall were the most exposed ones to parasitism [37]. Unraveling the relative importance of the different selective factors involved in the evolution of geographically differentiated fig wall thickness will prove challenging.

The process of diversification documented here could apply to numerous situations. Indeed within *Ficus*, intra-specific fig size variation is widespread and is spatially structured in at least some species [21, 22]. More generally, in many plant species, the size of some organs or structures, for instance the size of acorns [17], varies geographically and provides a series of situations in which processes analogous to the one documented here could apply.

## Conclusions

Non-pollinating fig wasp diversification has been shown to result from a series of processes including i) co-diversification with host plants [6, 35], ii) changes in ecological role within a community [35], iii) host plant shifts resulting in parasite speciation [35], and iv) diversification into parapatric species driven by adaptation to different ranges of variation in abiotic factors and competitive exclusion [8, 38]. In the situation documented here, diversification is driven by a biotic trait, geographic variation in host plant morphology. Hence, at least five different mechanisms of community diversification are at work in the wasp communities associated with figs.

Our results support the general hypothesis of an important role of biotic interactions in the diversification of life [46], and, in particular, the hypothesis of an important role for host plant phenotypic variation for the associated community constituted by phytophageous insect and their parasites. Because environmental conditions vary geographically, plants may vary in kind, resulting in different regional selection pressures on insects that attack the plants to diverge ecologically. This process occurs in fig wasp communities in conjunction with a series of other diversification processes, suggesting multiple answers to the question of why so many species.

## Additional file

Additional file 1: **Figure S1.** Neighbour joining COI phylogeny of 75 *Sycoscapter* individuals collected from *Ficus hirta*. **Figure S2.** Neighbour joining COI phylogeny of 70 *Philotrypesis* individuals collected from *Ficus hirta*. **Figure S3.** Assignation of microsatellite multilocus genotypes to cluster using STRUCTURE. **Figure S4.** Spatial genetic structure in *Ficus hirta* and its associated wasps. (PDF 252 kb)

## Abbreviations

COI: Cytochrome c oxidase subunit I
DNA: Deoxyribonucleic acid
*F*_*ST*_: *F*-statistics describe the statistically expected level of heterozygosity in a population. *F*_*ST*_ is the effect of subpopulations compared to the total population
He: Nei gene diversity corrected for sample size
K2P: Kimura 2-parameter pairwise genetic distance
PCR: Polymerase chain reaction
