# Supplementary material for: Plant geographic phenotypic variation drives diversification in its associated community of a phytophagous insect and its parasitoids
Source: BMC Evol Biol. 2018 Sep 4;18:134. doi: 10.1186/s12862-018-1239-5 (PMC6123920; doi:10.1186/s12862-018-1239-5)
Supplement: Supplementary file 1 — Figure S1. Neighbour joining COI phylogeny of 75 Sycoscapter individuals collected from Ficus hirta. Figure S2. Neighbour joining COI phylogeny of 70 Philotrypesis individuals collected from Ficus hirta. Figure S3. Assignation of microsatellite multilocus genotypes to cluster using STRUCTURE. Figure S4. Spatial genetic structure in Ficus hirta and its associated wasps. (PDF 252 kb) [file 12862_2018_1239_MOESM1_ESM.pdf]

Additional file 1. Plant geographic phenotypic variation drives diversification in its associated community of a phytophagous insect and its parasitoids

Figure S1. Neighbour joining COI phylogeny of 75 *Sycoscapter* individuals collected from *Ficus hirta*. *Philotrypesis* was used as outgroup. First letters designate sampling site, followed by haplotype number within site. All *Sycoscapter* sequences form a monophyletic clade that we interpret as a single species.

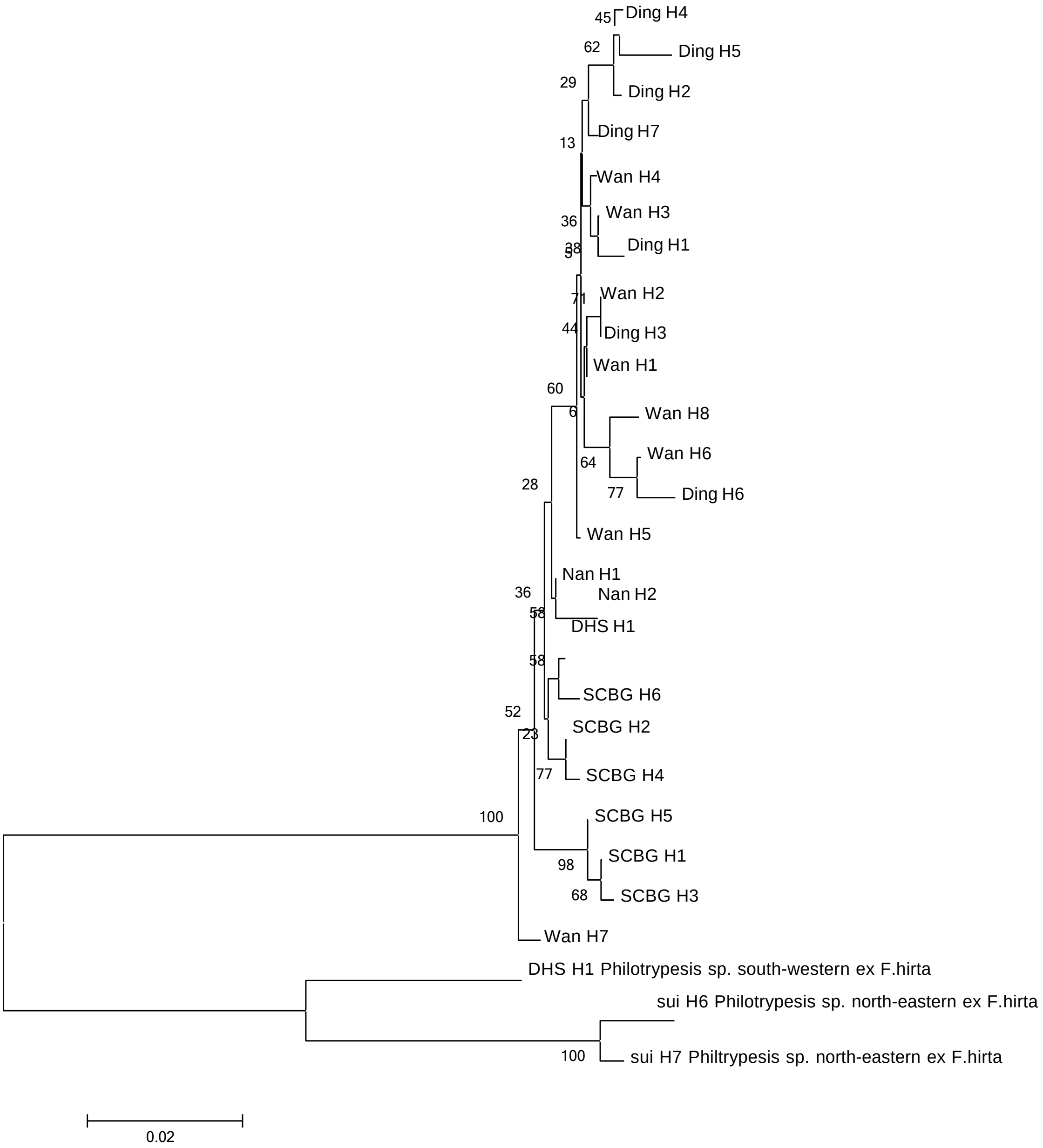

Figure S2. Neighbour joining COI phylogeny of 70 *Philotrypesis* individuals collected from *Ficus hirta*. *Philotrypesis* species associated with other subgenera of *Ficus* were used as outgroups. First letters designate sampling site, followed by haplotype number within site. Sequences of *Philotrypesis* from *Ficus hirta* form two monophyletic clades.

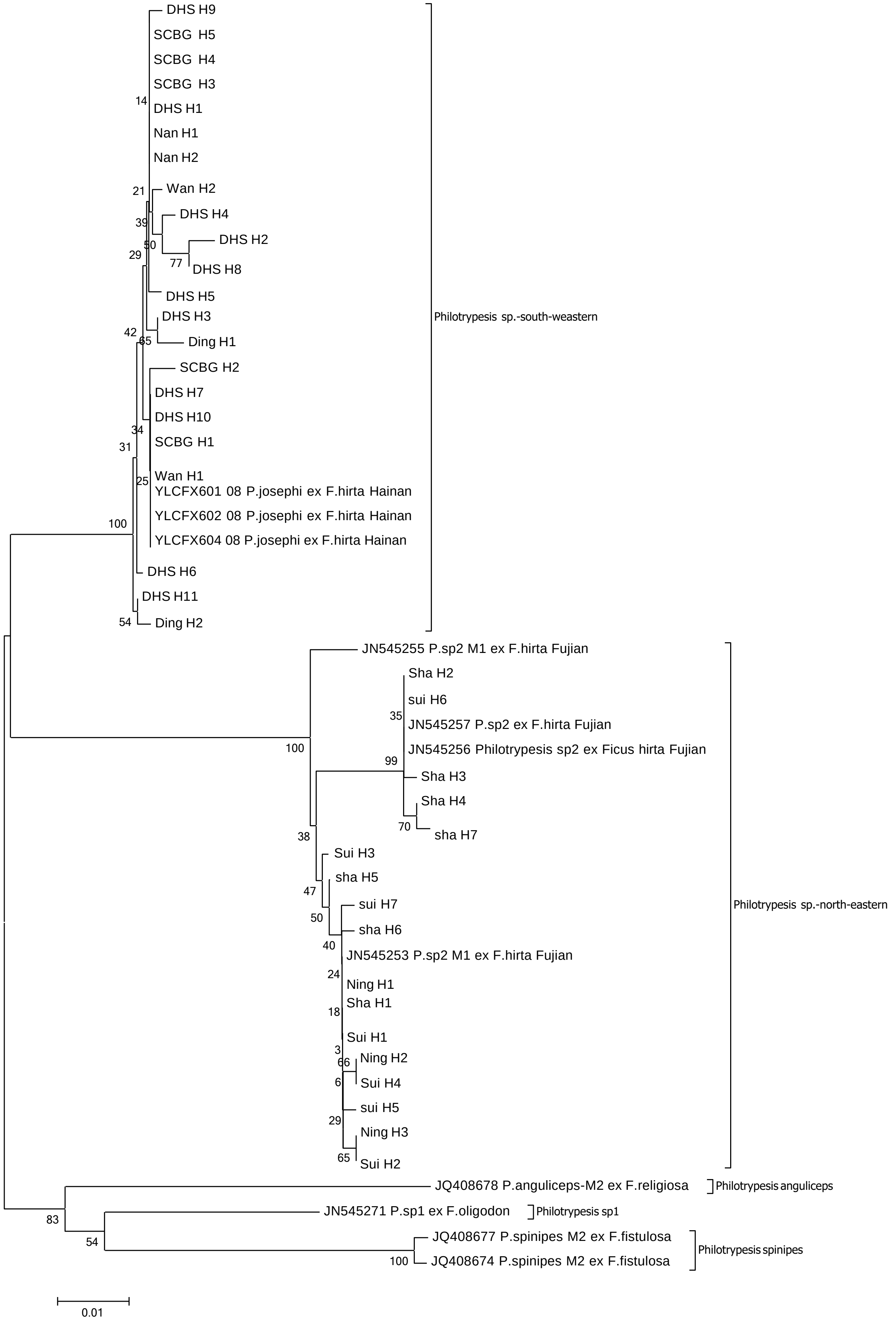

Figure S3

Assignation of microsatellite multilocus genotypes to cluster using STRUCTURE

a: *Sycoscapter*, K=2

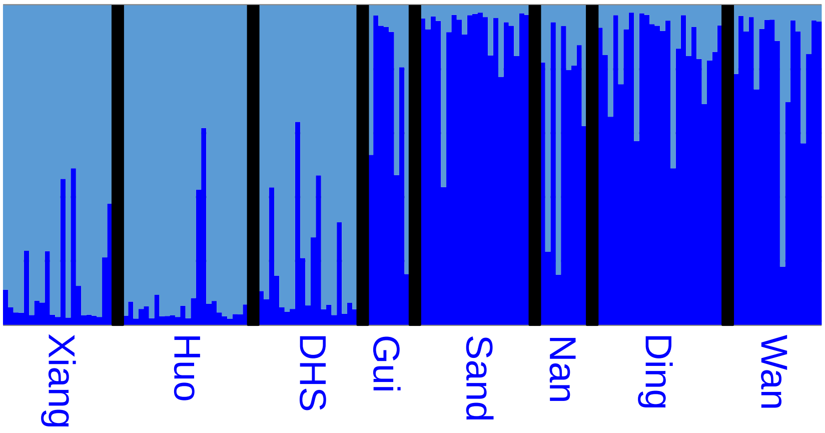

Genotypes from the closely located sites Xiang, Huo and DHS group together, suggesting spatial genetic structuring.

b: *Philotrypesis*, K=2

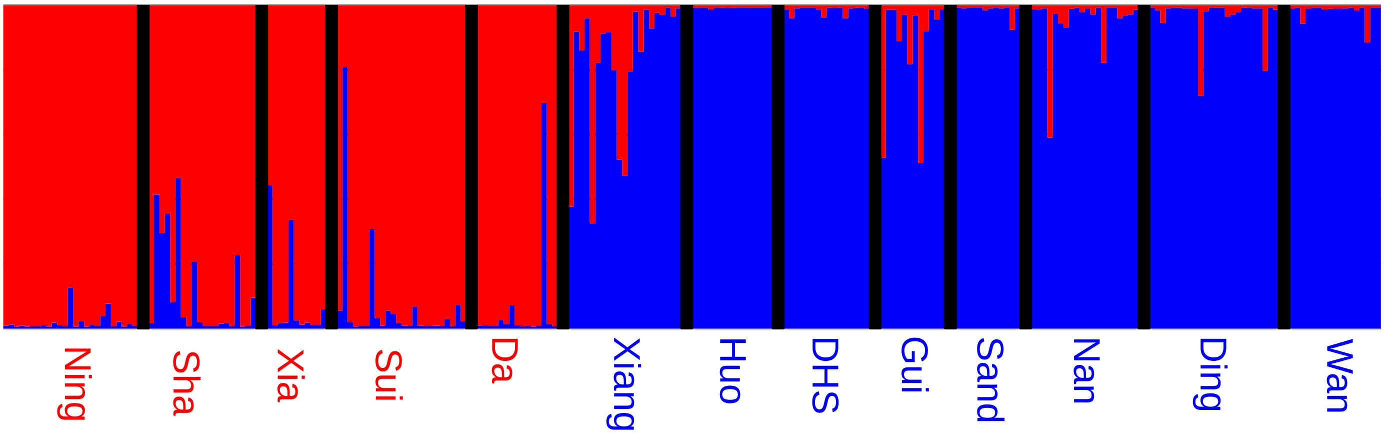

Genotypes from north-eastern locations (in red) are separated from genotypes from south-western locations (in blue), in agreement with the hypothesis of separation into two species.

c: *Philotrypesis*, K=4

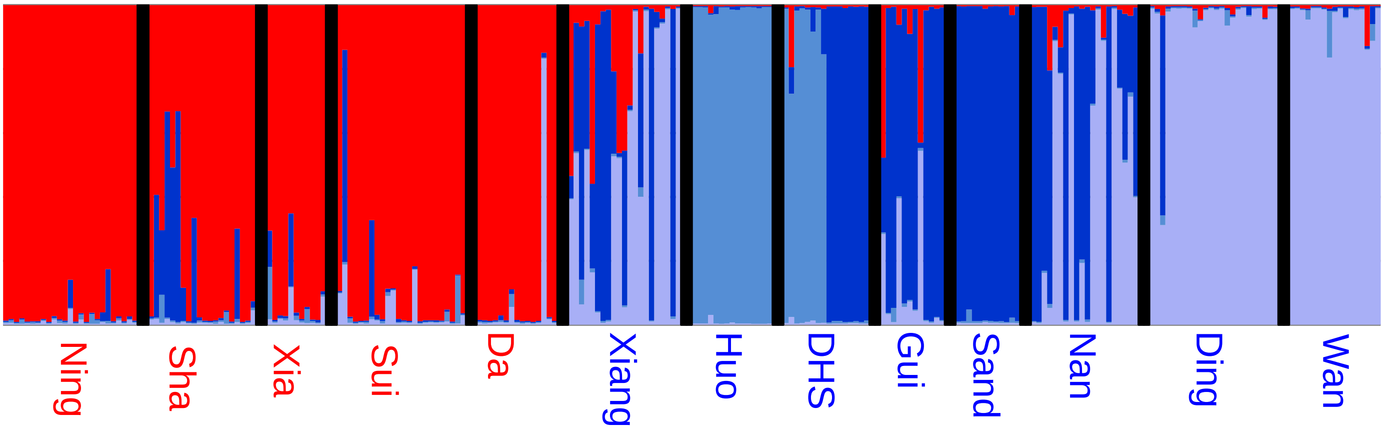

Genotypes from different south-western locations are separated into different clusters, in agreement with the high Fst values observed between populations.

Figure S4. Spatial genetic structure in *Ficus hirta* and its associated wasps

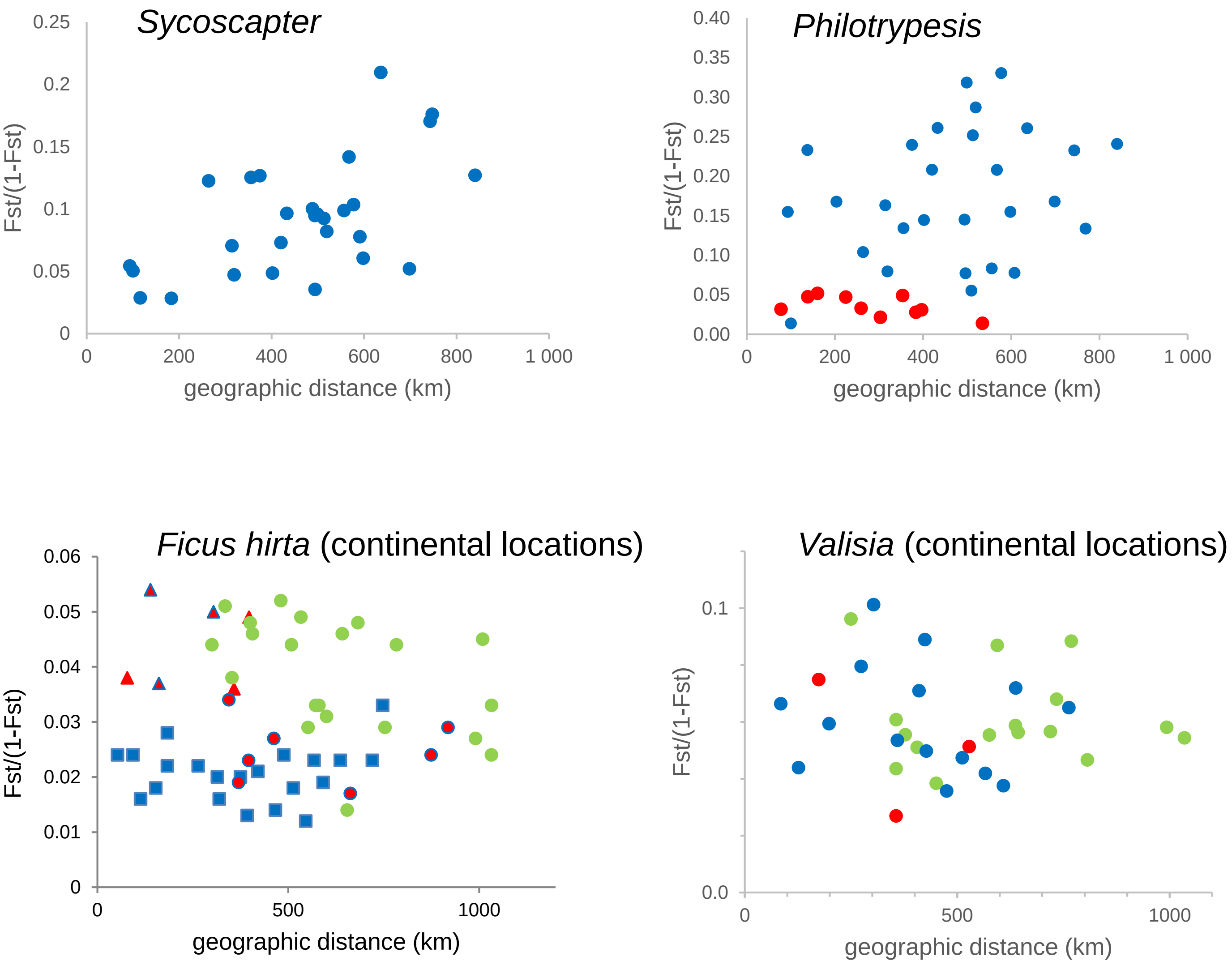

Microsatellite data.
Comparisons between south-western locations are in blue.
Comparisons between north-eastern locations are in red.
Comparisons between north-eastern and south-western locations are in green.

For *Ficus hirta*, comparisons between location Da (north-eastern population) and other locations are indicated in red with a blue margin. Rounds are for comparisons with south-western locations. Triangles for comparisons with north-eastern populations.

For *Sycoscapter* there is a pattern of genetic isolation by distance. For *Philotrypesis*, the north-eastern species is genetically very homogeneous while the south-western species presents strong unstructured differentiation between locations. For *Valisia*, genetic differentiation between populations is limited and there is no genetic isolation by distance. For *Ficus hirta*, the south-western locations and Da are highly homogeneous, while the three other north-eastern locations were more differentiated among themselve and comparatively to the other locations. The genetic differentiation of Hainan locations for *Ficus hirta* and *Valisia* (data not shown, Tian et al. 2015), is not observed in *Sycoscapter* and *Philotrypesis*.
